# Hearing Aid Effects and Satisfaction in Patients with Tinnitus

**DOI:** 10.3390/jcm11041096

**Published:** 2022-02-18

**Authors:** Hyun Jee Lee, Dae Woong Kang, Seung Geun Yeo, Sang Hoon Kim

**Affiliations:** Department of Otolaryngology—Head & Neck Surgery, School of Medicine, Kyung Hee University, Seoul 02447, Korea; hihyunz91@naver.com (H.J.L.); kkang814@naver.com (D.W.K.); yeo2park@gmail.com (S.G.Y.)

**Keywords:** tinnitus, hearing loss, hearing aid, counseling

## Abstract

This study aimed to evaluate the effectiveness of and satisfaction with hearing aids as a treatment option for tinnitus with hearing loss. Methods: This retrospective study used the tinnitus handicap inventory (THI), the satisfaction with amplification in daily life (SADL) questionnaire, and a medical chart review. A total of 116 patients treated between August 2018 and December 2020 were included. All patients with tinnitus and hearing loss underwent the same counseling sessions. Sixty patients chose to have hearing aids fitted (aided group), whereas 56 patients chose not to (non-aided group). Both the groups had similar audiometric configurations, durations of tinnitus, and ages. Structured interviews were performed, with various measures evaluated using the visual analog scale (VAS) and the THI questionnaire, before and six months after fitting the hearing aids. The SADL questionnaire was administered 6 months after fitting the hearing aids. Results: The patients’ THI scores reduced 6 months after the counseling, but the improvement in the THI scores was only significant in the group that received hearing aids. There were significant differences between the VAS scores of the two groups, and the changes in the VAS scores in the groups were statistically different. Subjective satisfaction with a hearing aid increased with improvements to tinnitus-related discomfort. Conclusion: The study’s results indicated that patients with hearing loss and tinnitus can be treated with hearing aids and counseling.

## 1. Introduction

Tinnitus is the perception of sound by a person without the presence of an external sound source. Various reasons can cause tinnitus, and its treatment is challenging because it has a deep association with the central nervous system and emotion. Acoustic therapy is important for treating tinnitus. This therapeutic method enables patients to decrease their recognition of tinnitus by increasing external auditory stimuli using a hearing aid or a sound generator [1]. Since several hearing-impaired patients complain of tinnitus, and because hearing aids are used for acoustic therapies, it is assumed that hearing-impaired patients with tinnitus are subjectively satisfied with their hearing aids, and that hearing aids can reduce tinnitus severity.

Hearing aid technology has developed significantly over the past years, but limited studies have investigated improvements in tinnitus following hearing aid use in patients with hearing difficulties. According to Surr et al., 62% of patients who began using a hearing aid had tinnitus, which declined in approximately half of them following hearing aid use [2]. However, there was no significant correlation between a degree of improvement in the tinnitus handicap inventory (THI) score after hearing aid use in patients with tinnitus and their satisfaction, measured with the Abbreviated Profile of Hearing Aid Benefit (APHAB)—a questionnaire that evaluates the performance of a hearing aid before and after its use [3]. This approach has a limited ability to evaluate patients’ subjective satisfaction associated with the benefits of using hearing aids prescribed for tinnitus-related hearing impairments. Various factors influence patient satisfaction, and subjective satisfaction is important in determining the success of prescription hearing aids. The Satisfaction with Amplification in Daily Life (SADL) questionnaire developed by Cox et al. [4] has 15 questions that evaluate four items: positive effects, negative features, service and cost, and personal image. SADL is useful for measuring patient satisfaction, including non-audiological elements and audiological elements that can be obtained after hearing aid use. We think that for tinnitus patients with hearing loss, counseling and hearing aid treatment is more effective than counseling alone. In addition, the degree of tinnitus improvement with hearing aids may be related to a patient’s satisfaction with their hearing aids.

Therefore, this study aimed to (1) compare improvements in the THI scores of patients who choose hearing aids with counseling and in those who choose only counseling and to (2) investigate the association between improvements in tinnitus and subjective patient satisfaction with hearing aids after the tinnitus improvements based on the THI score and the SADL questionnaire.

## 2. Materials and Methods

### 2.1. Patients

Our data were obtained from patients with bothersome tinnitus and hearing loss treated at the tinnitus clinic in our medical center between August 2018 and December 2020; 60 patients were treated with hearing aids and counseling (aided group), and 56 patients were prescribed only counseling (non-aided group). No information about this study was provided to the patients. Patients were excluded if they received other treatments, used a combination of devices, experienced other potential confounding injuries (e.g., noise trauma or a stroke) during the study, had a low THI score (<15) at their first visit, or did not use their hearing aids as recommended. Patients with vertigo were also excluded from our study, and patients with asymmetrical sensorineural hearing loss received an MRI to exclude other diseases, such as vestibular schwannoma. Tinnitus etiologies were distinguished from other middle-external ear diseases and Meniere’s disease through otolaryngological and neurological examinations. No patients had ever received counseling as a treatment, and none had previously used hearing aids. The study protocol was approved by the Institutional Review Board of Medical Center (IRB No. 2019-07-065).

### 2.2. Procedure

This study was a retrospective, questionnaire-based evaluation of treatment success. All first-visit patients underwent audiological evaluations. Pure-tone audiometry was performed at a frequency range of 0.25–8 kHz in a sound-proof room using an interacoustic AC40 audiometer. BC audiometry was performed at 0.5, 1, 2, and 4 kHz frequencies. Speech discrimination scores were calculated by estimating the percentage of correct word recognition out of a list of 50 phonetically balanced words, which was presented at 40 dB louder than the speech reception threshold. All the patients in the study were tested using audiological evaluations, REM, and hearing aid fittings performed by two clinicians with extensive experience. All the patients received counseling, and the test results were explained. As a treatment option, amplification was recommended for each patient. In the study, the counseling group chose not to use hearing aids or other sound therapy devices. Counseling was provided by an otologist with extensive experience in tinnitus management. Counseling was performed based on the Jastreboff tinnitus model and treatment strategy. The counseling can be summarized as follows: (1) an explanation of tinnitus’s origins, (2) a description of the tinnitus evaluation and hearing test, (3) a resolution of questions and anxiety about tinnitus, and (4) an explanation of the treatment course and goal of tinnitus management.

### 2.3. Hearing Aid Selection and Fitting

Hearing aid selection depended on personal preference and audiometric configuration. All the hearing devices used in this study were digital hearing aids. For patients who had unilateral hearing loss with tinnitus, we recommended a unilateral hearing aid on their affected side. Two hearing aids were recommended if the patient had bilateral hearing loss. When patients with bilateral hearing loss could only use unilateral hearing aids due to personal preferences or economic conditions, a unilateral hearing aid was applied to the side where tinnitus sounds were louder. The audiometric profile typical of most people suggested tinnitus at normal low frequencies with mild to moderate high-frequency SNHL; thus, hearing aids with open-fitting slim tubes were frequently selected. Every hearing aid was optimized for the amplification of low-input sounds. Real ear probe microphone measurements (REM, Aurical plus, GN Otometrics, Copenhagen, Denmark) were performed for all hearing aids to support optimal match prescriptions, and the prescription method was NAL-NL2. During the fitting period, the devices were further tuned according to subjective reports. For tunings, follow-up visits were scheduled based on each patient’s requirements. The hearing aid group was required to use their devices maximally, mostly in quiet environments where the tinnitus was very clear, even when communication was not a high priority.

### 2.4. THI and SADL

The self-administered THI questionnaire was used to quantify the impacts of tinnitus on individuals. They were classified as emotional stress, functional disorders, and disaster thoughts. The questionnaire had 25 questions, and each had the following responses to choose from: “Not so”, “Sometimes it is so”, and “It is so”, which were scored as 0, 2, and 4 points, respectively. We measured THI scores before either the counseling or hearing aid fittings and 3 months after the first THI measurement.

For the satisfaction investigation, we used the SADL scale, which consisted of 15 questions covering four items (positive effects, negative aspects, service and cost, and self-image) 6 months after hearing aid use commenced. The SADL scale, which consists of a global score (a mean value of these four items), was used, ranging from ”not at all” (1 point) to “always so or very much so” (7 points). One of these values was selected for scoring. The SADL investigation was performed three months after the hearing aid fittings. We conducted a follow-up fitting every month. The total VAS score was obtained based on the following question: “How loud or strong was your tinnitus on average over the last month?” The score ranged from 0 to 10 (0, no tinnitus; 10, as loud as you can imagine).

### 2.5. Statistical Analysis

The data were analyzed using SPSS software (version 19.0, IBM Corp., Armonk, NY, USA). Student’s *t*-test was used to compare the demographic data and VAS scores for tinnitus in the groups. The chi-square test was used to evaluate the effects of hearing aids on tinnitus according to changes in THI scores. The total changes in and classified THI scores of the two groups and the changes in their THI and SADL scores according to the fitting sites and the locations of tinnitus were analyzed with the Mann–Whitney test. The changes in the THI scores and the overall SADL scores were assessed using Spearman’s correlation test. Statistical significance was set at *p* < 0.05.

## 3. Results

Table 1 lists the patient characteristics obtained at the initial tinnitus clinic appointments. No preselection was performed to balance the two groups based on the participants or tinnitus or hearing features. The demographic data and tinnitus characteristics of the two groups showed no significant differences (Table 1).

All the patients had sensorineural hearing loss. In most cases, the patients had mild symmetrical high-frequency gently sloping hearing loss. The two groups showed no significant differences across the frequencies tested between 250 Hz and 8000 Hz for both ears (Table 2).

**Figure 1:**
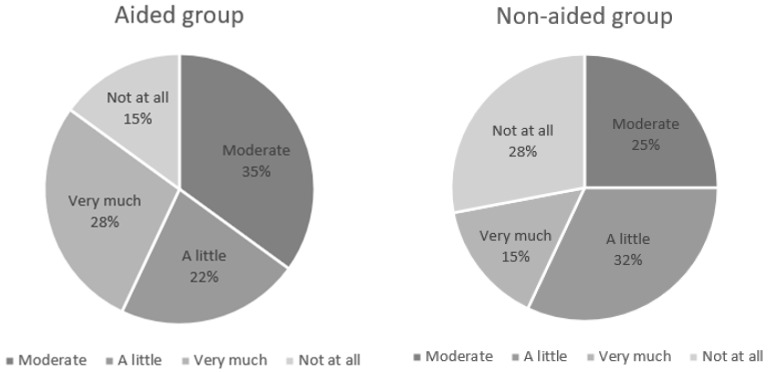
Distributions of the usefulness of wearing hearing aids for tinnitus according to the changes in the THI scores. (Aided group, hearing aids with counseling; non-aided group, no hearing aids (counseling only)).

The differences between the THI scores obtained before and after wearing a hearing aid and receiving counseling (aided group) or receiving only counseling (non-aided group) were categorized as follows: >30 points, very much; 10–30 points, moderate; 1–10 points, a little; and <1 point, not at all. In the aided group, 51 (85%) patients reported that the hearing aids were “helpful to some degree”, and 9 (15%) reported that they were “not helpful at all”; these numbers for the non-aided group were 41 (73%) and 15 (28%), respectively (Figure 1).

**Figure 2:**
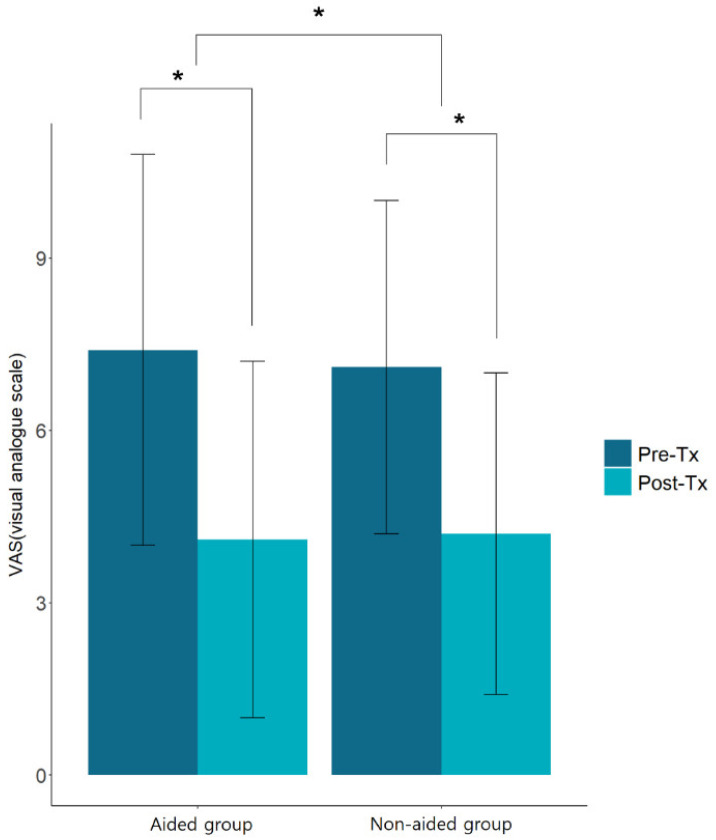
Comparison of the VAS (visual analogue scale) scores of the aided group and non-aided group. * *p* < 0.05 (Aided group, hearing aids with counseling; non-aided group, no hearing aids (counseling only)).

**Figure 3:**
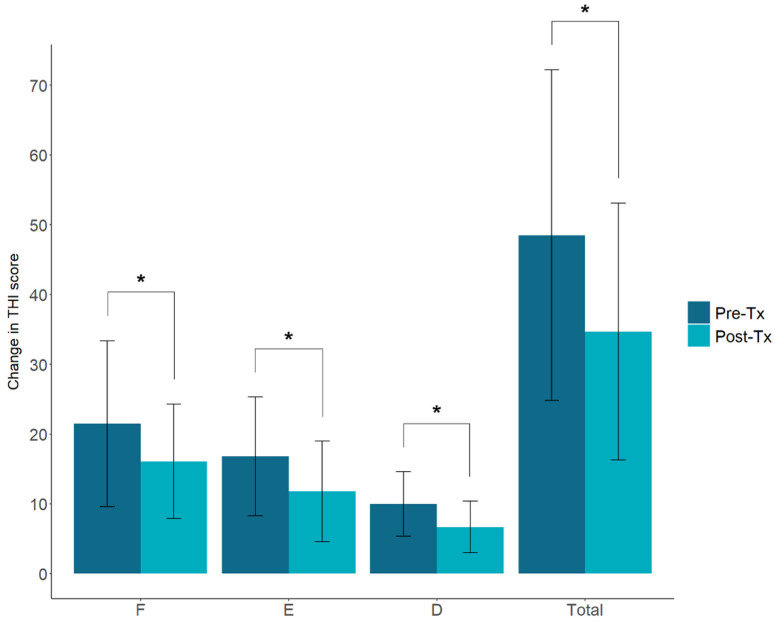
Comparative graph of the changes in the THI scores of the aided group. * *p* < 0.05 (F, functional; E, emotional; D, disaster thoughts).

Each group showed significant improvements in the VAS scores, and the changes in the VAS scores of both groups (aided group, 7.4 ± 3.4 to 4.1 ± 3.1; non-aided group, 7.1 ± 2.9 to 4.2 ± 2.8) were significant (*p* < 0.05; Figure 2). The comparison of the THI scores obtained before and after the patients wore hearing aids showed a decrease from 21.5 ± 11.9 to 16.1 ± 8.2 points for functional disorders (F), 16.8 ± 8.5 to 11.8 ± 7.2 points for emotional stress (E), 10.0 ± 4.6 to 6.7 ± 3.7 points for disaster thoughts (C), and 48.5 ± 23.7 to 34.7 ± 18.4 points for the total score; all the values were statistically significant (*p* < 0.05; Figure 3).

Forty-one patients wore unilateral hearing aids (unilateral tinnitus = 29; bilateral tinnitus = 12), whereas 19 wore bilateral hearing aids (unilateral tinnitus = 11; bilateral tinnitus = 8). Table 3 shows the effects of hearing aids on patients who were prescribed bilateral and unilateral aids and the patients’ satisfaction. Between the changes in the THI and SADL scores of the monaural and binaural hearing aid groups, there were no significant differences (*p* = 0.72; Table 3).

**Figure 4:**
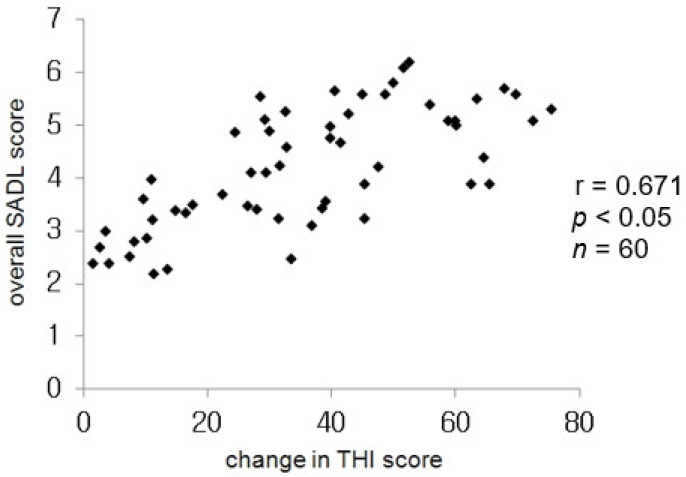
Correlation between the changes in the THI scores and the overall SADL score.

**Figure 5:**
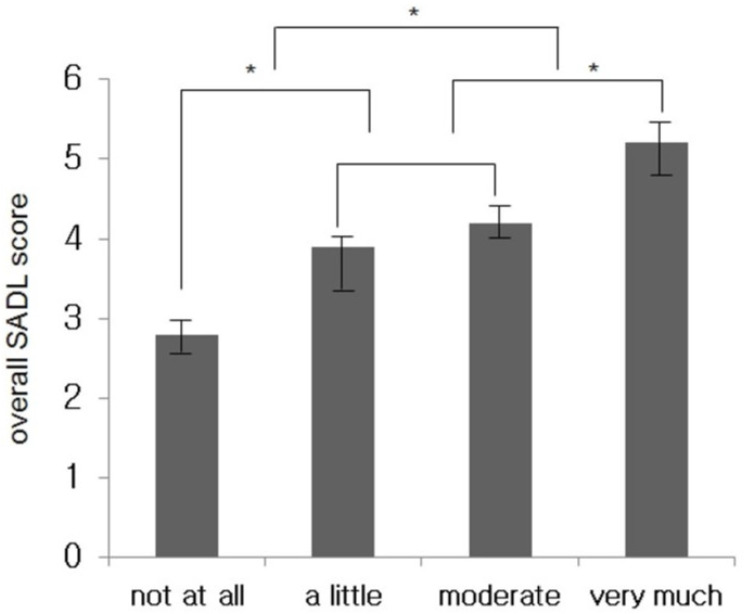
Comparative graph of the SADL scores according to the usefulness of wearing hearing aids for tinnitus. * *p* < 0.05.

The changes in the THI and overall SADL scores showed a positive correlation and were statistically significant (r = 0.671, *p* < 0.05; Figure 4). Regarding the effects of hearing aids on the aided group, there was no significant difference between groups that experienced small and moderate effects, but there were significant differences between the other groups (Figure 5).

## 4. Discussion

Tinnitus accompanied by sensorineural hearing loss is, in most cases, difficult to cure. Although a definite treatment for most chronic tinnitus cases is unavailable, patients can obtain symptom relief with assistance from informed clinicians. Tinnitus management is not aimed at masking or removing patients’ physical perceptions of tinnitus. To successfully manage tinnitus, clinicians should help patients pay less attention to the tinnitus. The ultimate aim of tinnitus management is to reduce the severity of tinnitus. The severity of tinnitus can be defined in several ways: how often or how much tinnitus bothers patients, how often tinnitus impairs patients’ lives, and how much patients suffer from the conditions induced by tinnitus [5].

In 1947, Saltzman et al. first reported that hearing aids benefit patients with tinnitus [6], a result that was subsequently confirmed by other studies [7,8]. Recently, Yokota et al. reported that when using hearing aids, acoustic therapy is useful for tinnitus. Unilateral hearing aids can improve tinnitus, as can bilateral hearing aids [9].

Surr et al. reported that THI administrations before and after hearing aid fittings indicate a statistically significant improvement of tinnitus based on THI scores measured only 6 weeks after the hearing aids are fitted; they also reported that 90% of patients with tinnitus may benefit from hearing aid amplification [3]. Forti et al. reported the efficacy of open-canal fittings for tinnitus with mild hearing loss patients [10]. Recently, Yakunina et al. reported that hearing aids effectively suppress tinnitus in patients with tinnitus along with high-frequency hearing disturbances [11]. However, a meta-analysis could not demonstrate any significant results following hearing aid use in patients with tinnitus [12].

The rationale underlying tinnitus treatments with hearing aids is based on two complementary hypotheses. Most types of tinnitus occur in conjunction with mild hearing disturbances or high-frequency hearing loss [13]. Hearing disturbances cause a lack of auditory input that can lead to tinnitus through the expression of neural plasticity and can cause the auditory pathway’s neurons to be hyperactive [14]. Hearing aids are beneficial because they can rehabilitate sound input and stimulate cerebral plasticity [15]. It is possible to stimulate cerebral plasticity and, at least partly, re-establish the adequate functioning of the auditory nerve pathways, which limits one of the probable causes of tinnitus. Hearing aid amplification increases neural activity, whereas tinnitus is worsened by silence; the brain may look for neural stimulation, which is attenuated secondary to hearing loss. Insufficient neural inhibition may be involved with tinnitus, and hearing aid amplification may facilitate the autocorrection of the brain’s inhibitory function [16].

Hearing aids are effective at reducing tinnitus severity perceptions, providing continuous sound stimulation. Hearing aids amplify background noise to potentially provide partial masking while decreasing the loudness or prominence of tinnitus. Hearing aids can be useful for habituation to the perception of tinnitus noise. Thus, tinnitus gradually becomes less annoying and reduces listening fatigue and stress with subsequent improvements to the coping abilities of patients. Hearing loss patients with hearing aids can potentially reduce the effects of tinnitus indirectly by improving communication, reducing stress and anxiety. Individuals with both untreated tinnitus and untreated hearing loss are expected to have an even more diminished quality of life than people with either tinnitus or hearing loss alone. We think hearing aid use may have a double impact on improving the quality of life of people with hearing loss and tinnitus.

Counseling is the basis of most tinnitus treatments [17]. In most contexts of audiology, counseling involves psycho-education on the physiological and psychological basis of tinnitus and simple plans to counter its effects. Hallam et al. reported that tinnitus patients have to adjust to the perception of internal noise; moreover, they often have to adjust to the negative feelings and consequences that follow it [18]. Counseling that adequately explains the relationship between tinnitus and hearing disturbances, as well as the proper use of hearing aids, can help tinnitus and hearing loss.

Many patients with tinnitus do not recognize that they have hearing loss. Patients with tinnitus report that they strain to hear adequately, which affects their concentration, increases their stress levels, and promotes annoyance.

Patients with hearing loss have higher incidences of increased stress than people with normal hearing [19]. Relieving these components via hearing aid use can benefit individuals with hearing impairments. Therefore, individuals with both untreated tinnitus and untreated hearing loss are expected to have an even more diminished quality of life than individuals with either tinnitus or hearing loss alone.

Tinnitus is an auditory disorder that is heavily influenced by stress. The majority of people who suffer from persistent tinnitus have some degree of hearing loss. Current theories about the origins of tinnitus and the associated emotional distress suggest that the perception of tinnitus may result, at least partly, from the peripheral attenuation of auditory input; this attenuation increases central auditory system activity from the dorsal cochlear nucleus to the auditory cortex and couples with the limbic system via collateral connections with the thalamus and other structures [20]. Thalamic stimulation results in the release of neurotransmitters, including epinephrine, to produce an autonomic nervous system response associated with stress [21].

In this study, 85% of the patients reported an improvement in tinnitus after wearing a hearing aid and receiving counseling, whereas counseling alone improved tinnitus in 73.3% of the patients. When comparing the items on the THI questionnaire, all the items—functional disorders, emotional stress, and disaster thoughts—showed significant improvements. According to a recent review article, positive results of wearing hearing aids for tinnitus relief were shown by 68% of studies, whereas 14% demonstrated no change in tinnitus perception [22]. These results are similar to those of our study.

This study was limited by its relatively short-term follow-up period that was insufficient for predicting the effectiveness of hearing aids and counseling. However, despite the short-term follow-up results, the symptoms of tinnitus improved significantly; therefore, this study is significant for patients with sensorineural hearing loss with tinnitus as it provides an initial treatment approach. Further research, including a long-term follow-up on hearing aids and counseling, is required for patients with tinnitus.

In addition, the degree of improvement in tinnitus between the unilateral and bilateral hearing aid users showed no significant difference. However, this could have been the result of a decrease in the number of patients due to the economic burden of purchasing a bilateral hearing aid. If possible, hearing aids should be prescribed for both the ears as this enables a better understanding of verbal messages and spatial localization. These two elements are important in activating the entire acoustic nerve system [21]. Further research is required to determine whether such a mechanism is involved in patients who show improvements in THI scores after wearing hearing aids on the side contralateral to the tinnitus-affected side.

The success of hearing aid use depends on the extent of improvement in a patient’s ability to have a conversation in daily life and the outcomes of hearing tests. Therefore, several questionnaires have been designed to determine the benefits of hearing aids. The Hearing Aid Profile Inventory (HAPI), HHI, and APHAB are well-known questionnaires that investigate the effects of hearing aids [23,24,25]. However, these evaluation methods have limitations because they assess only the benefits of communication and do not include various variables that can yield positive results for patient satisfaction with a hearing aid. The SADL scale, developed by Cox et al. [4] is a simple questionnaire that can evaluate various elements of satisfaction independently. Translated versions of the SADL have been validated as useful tools for measuring satisfaction with hearing aids and fittings in Korea [26]. Various variables of satisfaction are summed up in four items and 15 questions in various questionnaires and investigations. The four items consist of positive effects that reflect audiological benefits and psychological satisfaction; negative features that reflect surrounding noises, reechoes, and telephone use; service and cost; and self-image, which reflects a cosmetic aspect. Veiga et al. reported that patients with sensorineural hearing loss and tinnitus or recruitment have difficulty in understanding auditory information despite hearing aid use, especially in a noisy environment [27]. Similarly, patients with sensorineural hearing loss and tinnitus show relatively lower effects of acoustic amplification. However, in this study, the SADL score correlated significantly with the THI score; therefore, we consider that the degree of improvement in tinnitus is associated with a patient’s satisfaction with a hearing aid.

We had several limitations in this study. Patients were not randomly assigned in this study, which was conducted through a chart review as a retrospective study. As a result, there may have been a selection bias in the sample selection process. Rachel et al. recently published a randomized controlled trial study in this field [11,28]. Moreover, the physician and the participants could not be blinded to the treatment allocations because hearing aids were given to only the intervention group. Furthermore, the possibility of a placebo effect in the hearing aid patients could not be totally eliminated in this research. According to Hoare DJ et al., there is likely to be a potential for placebo effects when treating tinnitus, and psychosomatic symptoms, such as stress or anxiety, can influence the outcome [29]. In the future, we intend to conduct a randomized controlled trial study on the use of hearing aids to treat tinnitus.

## 5. Conclusions

It is expected that the prescription of hearing aids for patients with hearing impairments and tinnitus can provide audiological benefits and can positively impact tinnitus treatments. In addition, the THI can be used to evaluate the degree of an audiological improvement to predict and evaluate success following hearing aid use in such patients, and the SADL questionnaire is helpful for measuring satisfaction by considering various variables.

## Figures and Tables

**Table 1:** Characteristics of patients at their initial tinnitus clinic appointments.

	Aided Group (*n* = 60)	Non-Aided Group (*n* = 56)	*p* Value
Age (years)	52.8 ± 13.1	49.8 ± 12.2	0.344
Gender (M/F)	28/32	33/23	0.542
Years from tinnitus onset	5.1 ± 2.3	4.1 ± 3.5	0.376
Rt side mean PTA (dB)	35.7 ± 22.2	33.8 ± 33.8	0.437
Lt side mean PTA (dB)	37.7 ± 23.8	34.1 ± 23.3	0.284
Unilateral tinnitus	40	38	0.237
Bilateral tinnitus	20	18	0.704
VAS	7.4 ± 3.4	7.1 ± 2.9	0.667
THI	66.5 ± 24.1	54.3 ± 27.4	0.219
SDS	74 ± 24	72 ± 23	0.805

VAS, visual analogue scale; THI, tinnitus handicap inventory; SDS, speech discrimination score; mean PTA, ((500 Hz + 2 × 1000 Hz + 2 × 2000 Hz + 4000 Hz)/6); aided group, hearing aids with counseling; non-aided group, no hearing aids (counseling only). *p* value was calculated using Student’s *t*-test.

**Table 2:** Averaged PTA conduction thresholds (dB HL) at the initial visits.

Hz	Aided Group (*n* = 60)		Non-Aided Group (*n* = 56)		*p* Value
	Right (dB)	Left (dB)	Right (dB)	Left (dB)	
250	26.6 ± 18.1	28.6 ± 14.1	21.1 ± 18.1	22.4 ± 12.1	0.544
500	26.7 ± 19.2	27.7 ± 16.2	23.7 ± 19.2	24.8 ± 13.1	0.352
1000	30.2 ± 21.1	33.2 ± 23.3	27.2 ± 14.8	25.8 ± 15.4	0.256
2000	35.2 ± 22.2	36.2 ± 27.9	33.3 ± 18.2	36.8 ± 13.7	0.335
3000	52.8 ± 22.9	57.8 ± 27.7	49.8 ± 25.9	51.4 ± 22.1	0.263
4000	56.8 ± 22.6	59.8 ± 24.2	58.2 ± 23.6	54.6 ± 33.1	0.607
8000	63.3 ± 24.5	68.3 ± 29.2	64.3 ± 25.7	61.8 ± 28.3	0.523

Aided group, hearing aids with counseling; non-aided group, no hearing aids (counseling only). *p* value was calculated using Student’s *t*-test.

**Table 3:** Changes in the THI and SADL scores according to the fitting sites and locations of tinnitus.

Side of Hearing Aid	Site of Tinnitus	Change in THI Score	SADL Score
Monoaural (*n* = 41)	Unilateral (*n* = 29)	34.3 ± 25.6	4.83 ± 0.94
	Bilateral (*n* = 12)	32.8 ± 24.4	4.61 ± 1.12
Biaural (*n* = 19)	Unilateral (*n* = 11)	38.2 ± 22.8	4.84 ± 0.89
	Bilateral (*n* = 8)	36.3 ± 23.2	5.04 ± 0.73

## References

[1] Pienkowski M. (2019). Rationale and efficacy of sound therapies for tinnitus and hyperacusis. Neuroscience.

[2] Surr R.K., Montgomery A.A., Mueller H.G. (1985). Effect of amplification on tinnitus among new hearing aid users. Ear Hear..

[3] Surr R.K., Kolb J.A., Cord M.T., Garrus N.P. (1999). Tinnitus Handicap Inventory (THI) as a hearing aid outcome measure. J. Am. Acad. Audiol..

[4] Cox R.M., Alexander G.C. (1999). Measuring satisfaction with amplification in daily life: The SADL scale. Ear Hear..

[5] Folmer R.L., Martin W.H., Shi Y. (2004). Tinnitus: Questions to reveal the cause, answers to provide relief. J. Fam. Pract..

[6] Saltzman M., Ersner M.S. (1947). A hearing aid for the relief of tinnitus aurium. Laryngoscope.

[7] Kiessling J. (1980). Masking of tinnitus aurium by maskers and hearing aids (author’s transl). HNO.

[8] Miller M. (1981). Tinnitus amplification: The high frequency hearing aid. J. Laryngol. Otol. Suppl..

[9] Yokota Y., Yamashita A., Koyama S., Kitano K., Otsuka S., Kitahara T. (2020). Retrospective evaluation of secondary effects of hearing aids for tinnitus therapy in patients with hearing loss. Auris Nasus Larynx.

[10] Forti S., Crocetti A., Scotti A., Costanzo S., Pignataro L., Ambrosetti U., Del Bo L. (2010). Tinnitus sound therapy with open ear canal hearing aids. Acta Oto-Rhino-Laryngol. Belg..

[11] Yakunina N., Lee W.H., Ryu Y.J., Nam E.C. (2019). Tinnitus suppression effect of hearing aids in patients with high-frequency hearing loss: A randomized double-blind controlled trial. Otol. Neurotol..

[12] Sereda M., Xia J., El Refaie A., Hall D.A., Hoare D.J. (2018). Sound therapy (using amplification devices and/or sound generators) for tinnitus. Cochrane Database Syst. Rev..

[13] König O., Schaette R., Kempter R., Gross M. (2006). Course of hearing loss and occurrence of tinnitus. Hear. Res..

[14] Kaltenbach J.A. (2006). Summary of evidence pointing to a role of the dorsal cochlear nucleus in the etiology of tinnitus. Acta Oto-Laryngol..

[15] Gabriel D., Veuillet E., Vesson J.F., Collet L. (2006). Rehabilitation plasticity: Influence of hearing aid fitting on frequency discrimination performance near the hearing-loss cut-off. Hear. Res..

[16] Sweetow R.W., Sabes J.H. (2010). Effects of acoustical stimuli delivered through hearing aids on tinnitus. J. Am. Acad. Audiol..

[17] Tyler R.S., Oleson J., Noble W., Coelho C., Ji H. (2007). Clinical trials for tinnitus: Study populations, designs, measurement variables, and data analysis. Prog. Brain Res..

[18] Hallam R., Rachman S. (1984). Psychological Aspects of Tinnitus in Contributions to Medical Psychology.

[19] Fellinger J., Holzinger D., Dobner U., Gerich J., Lehner R., Lenz G., Goldberg D. (2005). Mental distress and quality of life in a deaf population. Soc. Psychiatry Psychiatr. Epidemiol..

[20] Kaltenbach J.A., Zhang J., Finlayson P. (2005). Tinnitus as a plastic phenomenon and its possible neural underpinnings in the dorsal cochlear nucleus. Hear. Res..

[21] Del Bo L., Ambrosetti U. (2007). Hearing aids for the treatment of tinnitus. Prog. Brain Res..

[22] Jacquemin L., Gilles A., Shekhawat G.S. (2021). Hearing more to hear less: A scoping review of hearing aids for tinnitus relief. Int. J. Audiol..

[23] Cox R.M., Alexander G.C. (1995). The abbreviated profile of hearing aid benefit. Ear Hear..

[24] Walden B.E., Demorest M.E., Hepler E.L. (1984). Self-report approach to assessing benefit derived from amplification. J. Speech Lang. Hear. Res..

[25] Newman C.W., Weinstein B.E. (1988). The Hearing Handicap Inventory for the Elderly as a measure of hearing aid benefit. Ear Hear..

[26] Kim G., Lee E., Kim D., Kim J. (2018). An analysis study of features for hearing aid satisfaction questionnaires: Satisfaction with amplification in daily life, client oriented scale of improvement, international outcome inventory for hearing aids. Audiol. Speech Res..

[27] Veiga L.R., Merlo Á.R., Mengue S.S. (2005). Satisfaction level with hearing aid in the daily life of Army Healthcare System users. Rev. Bras. Otorrinolaringol..

[28] Haines R.H., White J., Meakin G., Tan W., Hepburn T., Leighton P., Theriou C., Stockdale D., Almey C., Nicholson R. (2020). Protocol for a multi-centre randomised controlled stand-alone feasibility trial to assess potential effectiveness and cost-effectiveness of digital hearing aids in patients with tinnitus and hearing loss (the HUSH trial). Pilot Feasibility Stud..

[29] Hoare D.J., Edmondson-Jones M., Sereda M., Akeroyd M.A., Hall D. (2014). Amplification with hearing aids for patients with tinnitus and co-existing hearing loss. Cochrane Database Syst. Rev..

